# Experimental Study on the Bending Behaviour of GFRP Laminates Repaired with Stainless-Steel Wire Mesh

**DOI:** 10.3390/polym17172417

**Published:** 2025-09-05

**Authors:** Hamza Taş, Hasan Yavuz Ünal

**Affiliations:** 1 Department of Mechanical Engineering, Hasan Ferdi Turgutlu Faculty of Technology, Manisa Celal Bayar University, 45400 Manisa, Turkey; hamza.tas@cbu.edu.tr; 2 Department of Mechanical Engineering, Faculty of Engineering, Ege University, 35040 İzmir, Turkey

**Keywords:** patch repair, patch material, woven wire mesh, damaged laminate, glass fibre composite, three-point bending test

## Abstract

This study experimentally investigates the use of stainless-steel woven wire mesh (SSWWM) as a patch material for repairing damaged glass fibre-reinforced (GFR) composite laminates. The effects of several factors on the three-point bending (3PB) behaviour of the parent laminate were examined, including the repair method (the plugging of open hole and the external patch repair), the mesh count of the SSWWM, and the number of SSWWM layers. According to the findings, all parameters considered in this study play a pivotal role in 3PB behaviour. Employing SSWWM as a patch material can recover 66.02–129.2% of the undamaged 3PB failure load, depending on the repair method, mesh count of the SSWWM, and number of SSWWM layers. Overall, decreasing the mesh count and increasing the number of SSWWM layers and applying an external patch repair method yield better results in terms of failure load and patch efficiency. This can be attributed to the increased wire diameter, improved bending rigidity, and better load distribution over a wider area. The SSWWM bridges the damaged zone, ensuring effective load transfer between the patch and parent laminate while preventing crack propagation. Utilising SSWWM as a patch material provides a quick, reliable solution for damage scenarios in engineering applications.

## 1. Introduction

Fibre-reinforced epoxy matrix composites have a variety of applications in diverse fields, including the automotive, aerospace, sport equipment and defence industries [1,2]. Their widespread adoption is primarily due to their outstanding mechanical properties, such as their high tensile and flexural strength, interlaminar shear strength, elastic modulus, toughness, and excellent strength-to-weight ratio [3]. However, composite materials are susceptible to damage during service. This damage may be caused by various factors, including environmental exposure, ageing-related degradation, and impact loading.

Several studies have been conducted to determine the long-term performance of composites under harsh environmental conditions. For instance, Yu et al. [4] investigated the water-absorption behaviour and interlaminar shear strength of GFRP bars. Their findings revealed that in GFRP bars immersed in water, the epoxy matrix swelled due to moisture absorption, generating hygrothermal stresses that could exceed the interfacial bond strength and cause interfacial cracking over time. Similarly, Öztürk et al. [5] attributed the reduction in the mechanical properties of GFRP composites subjected to water ageing to the degradation of the fibre–matrix interface. Furthermore, Ghabezi and Harrison [6] reported that water absorption can lead to hydrolysis of the polymer matrix, which in turn degrades the fibre–matrix interfacial bonding. In addition to environmental degradation, FRP composites are also vulnerable to accidental impact damage, such as tool drops or collisions with foreign objects. To address such issues and restore structural performance, patch repair techniques have been developed and employed [7,8].

Errahmane et al. [9] conducted a study to analyse the fatigue life of a cracked 2024-T3 aluminium plate that had been repaired using a carbon/epoxy composite material and a 2024-T3 aluminium patch, with and without the application of ageing. In both types of patches, the fatigue life of the specimen was increased by a factor of 10 compared to the unrepaired specimen. Moreover, the fatigue life of the aluminium-patched specimen was superior to that of the composite-patched specimen when water ageing was performed. Rajendran and Vellayaraj [10] also investigated the fatigue life of an aluminium specimen containing cracks. The carbon/epoxy composite patch containing nano-sized boron carbide demonstrated a significant reduction in plastic strain amplitude and energy dissipation, accompanied by an enhancement in fatigue life by a minimum factor of 5.1 in comparison with the unpatched specimen. In another study [11], the experimental and numerical investigation focused on the repair of carbon/epoxy composite specimens using conventional step-lap and novel I-lap patches. In the step-lap repair method, the plies of a composite laminate are cut and replaced in a stepped (overlapping) configuration, and the I-lap patch enhances this approach by forming a mortise-and-tenon-like interlocking joint between the patch and the parent material. It is evident that the high load-bearing capacity of the mortise and tenon structural design is responsible for the observed increase in tensile and flexural strength, which was recorded at 16% and 8%, respectively, when the I-lap patch was utilised as a repair method. The undamaged, drilled, and repaired tensile behaviour of hybrid composites in two different configurations was investigated by Sahoo et al. [12]. Eight different stacking sequence patches were used to cover the damage area. As a result of the investigation, the use of glass fibre patches showed the highest value in force recovery due to its lower stiffness in both hybrid composite specimens. Liu et al. [13] conducted an experimental and numerical investigation into the low-velocity impact behaviour of pristine and circularly patched composite specimens. Furthermore, impact tests were conducted by filling the hole area with a circular plug. The numerical and experimental results were found to be consistent with each other. The investigation by Messaoud and Meriem-Benziane [14] focused on the repair of semi-elliptically corroding pipeline steel using a composite material comprising jute fibre and steel wire. The steel wire/jute hybrid specimen was bonded to the pipeline specimen with three different damage depths/thicknesses (a/t) in single and double numbers, and bending tests were carried out. In the context of a/t = 0.2, it was found that the maximum force exerted by double patches exceeded that of an undamaged specimen.

A number of studies have been conducted by researchers to strengthen the joint surface and increase the adhesive strength. In addition to the utilisation of steel wire mesh to enhance adhesive strength, the studies have also encompassed the examination of metal components, fibres, and metal foams. Taş [15] examined the impact of the mesh count of stainless-steel woven wire mesh (SSWWM), SSWWM area, and SSWWM position on the joint strength of an aluminium adherend single-lap joint (SLJ). In the study, four different mesh sizes were used, and it was stated that reinforcement of the adhesive with SSWWMs increased the load-bearing capacity by approximately 46%. The utilisation of aluminium fibres in single-lap joints was the subject of an investigation by Khoramishad and Razavi [16]. The addition of metal fibres to the adhesive joint has been demonstrated to enhance the shear strength. Furthermore, the numerical analysis demonstrated that increasing the fibre diameter and decreasing the fibre distance resulted in a favourable stress distribution. Çalık et al. [17] experimentally and numerically investigated the effect of intermittent metal parts and countersunk metal parts on the joint strength of single-lap joints. As a result of the tests, it was stated that intermittent metal parts and countersunk metal parts increased the joint fracture load by 27% and 30%, respectively. In their study, Bayramoglu et al. [18] experimentally and numerically determined that the use of metal pieces with variable lengths in the bonding region increases the force bearing in SLJ between 17% and 41%. Kanani et al. [19] conducted a study to ascertain whether it would be possible to increase the strength of dissimilar SLJs made of polyphthalamide and aluminium alloy by metal reinforcement at the interface. In order to achieve this objective, the thickness and length of the patch were selected as variables and subjected to experimental and numerical investigation. The investigation revealed that the application of a 0.4 mm thick and 5 mm long patch resulted in an approximate 90% increase in joint strength compared to the unreinforced SLJ. Zhou and Chen’s investigation focused on the impact of porous metal foam on the shear strength and fatigue life of dissimilar (composite/aluminium) SLJs [20]. The addition of nickel foam to the adhesive resulted in a 67% increase in shear strength and 242%, 255%, and 1939% enhancements in mean fatigue life at 30%, 40%, and 50% stress levels, respectively. Furthermore, the incorporation of metal foam resulted in a modification of the crack propagation behaviour in dissimilar SLJ. The studies also include the use of pins and wires to strengthen adhesive joints [21,22]. Studies have shown that the homogeneous stress distribution of the metal wire and the bridging effect of the pin have a positive impact on the strength of the connection. Ravindran et al. [21] found that the use of z-pins in composite SLJ increased the joint strength by about 20%. Rezvaninasab et al. [22] conducted an experiment to determine whether using stainless-steel wires in the longitudinal direction and pins in the transverse direction would significantly increase joint strength compared to using adhesive alone. Furthermore, hybrid reinforcement (wires and pins together) increased maximum joint strength and toughness.

Steel wire mesh is a metallic material that is characterised by its cellular geometry. It is employed in a variety of structural applications, including impact-absorbing structures and cooling systems, as well as concrete reinforcement. The primary advantages of steel wire mesh are its low weight, high toughness, strength, and thermal conductivity [23,24,25]. In their study, Wang et al. [23] subjected steel wire mesh to low-velocity impact testing by clamping it in two different directions. In the study, the impactor mass, impact velocity, and impactor size were examined as variables. The results indicated that the specimens clamped from the weft wire exhibited a greater reaction force compared to those clamped from the warp wire. In the research conducted by Karunagaran et al. [26], glass fibre, in both its untreated and treated forms, was incorporated into a hybrid composite structure that integrated stainless-steel wire mesh. Consequently, the low-velocity impact test, which was conducted at three distinct impact velocities, demonstrated that the surface treatment applied to the glass fibre and steel wire mesh led to a substantial reduction in the absorbed energy. In the context of glass fibre/epoxy production, stainless-steel wire mesh was incorporated between layers to form a hybrid composite. In the study undertaken by Wan et al. [27], the static and dynamic properties of the hybrid plate were investigated through the alteration of the number of wire mesh layers. The utilisation of wire mesh at the maximum number of layers (five layers) resulted in a 34% and 38% increase in tensile and flexural strength, respectively, in comparison to the non-hybrid specimen. Additionally, the penetration threshold velocity and absorbed energy values were enhanced. Hasselbruch et al. [28] hybridised a carbon fibre/thermoplastic composite with steel wire mesh and investigated the effect of wire mesh and press force on tensile test results. As a result of the tests, it was reported that the strength and stiffness of the specimens with wire mesh were approximately 44% and 12% lower, respectively, compared to the carbon fibre-reinforced thermoplastic specimen for specimens manufactured under a 300-ton press. Furthermore, it was observed that the stiffness and strength of the hybrid specimens increased when the press force was reduced. In a related study, Truong et al. [29] examined the impact of the reinforcement of carbon fibre/epoxy composite with steel wire mesh on the material’s tensile properties. In addition to the number of steel wire meshes, the number of off-axis carbon fibre layers and the curing temperature were also considered as variables in the study. The findings of the test results indicate that steel wire mesh has a substantial impact on the initial stiffness, peak load, and residual load. Furthermore, steel wire mesh was utilised to mitigate the impact caused by wind. In the study by Meng et al. [30], steel wire mesh was placed on the Oriented Strand Board (OSB) surface or between the OSB surface and the polystyrene foam core. The objective of this study was to minimise wind damage to OSB. The findings of the test results indicated that the most advantageous approach was to position the steel wire mesh on the surface.

The widespread use of composite materials has attracted significant attention from researchers. Due to their susceptibility to damage during service life, effective repair strategies are essential. The literature reports various approaches to patching damaged composite structures, including the use of similar composite materials as well as metallic components such as aluminium and steel. It is of interest to clarify whether SSWWM can be used as a direct patch in composite structures. This study aims to address this gap in the existing literature. In this study, stainless-steel woven wire mesh (SSWWM) was utilised for the repair of GFR composite with a circular damage. The fabrication of unidirectional glass/epoxy composite plates was accomplished through the utilisation of the vacuum-assisted resin infusion method. From this plate, 77 mm×13 mm specimens with a thickness of 1.79 ± 0.02 mm and 5 mm diameter holes at their centre were obtained by using a water jet cutting machine. The circular shape hole was selected deliberately to reflect a common and practical approach in the repair of GFRP materials [31]. While a circular hole may not always represent the initial damage shape, it accurately reflects the post-processed damage condition commonly encountered during real-world repair scenarios. Three-point bending (3PB) tests were performed on damaged and patched specimens. The influence of plugging the open hole, external patch repair, SSWWM count, and number of SSWWM layers on the 3PB behaviour of damaged parent laminate were examined.

## 2. Experimental Details

### 2.1. Materials

In this work, unidirectional (UD) E-glass fabric with the areal density of 330 g/m^2^ (Product code: 03G330UD.127.01), supplied from Dost Kimya (İstanbul, Turkey), was used to fabricate glass fibre-reinforced (GFR) composite laminate, hereafter referred to as the parent laminate in the following sections. According to the supplier’s specs, the linear densities of the fibres in weft and warp directions are 68 tex (37 g/m^2^) and 1200 tex (283 gr/m^2^), respectively. Moreover, the linear density of the stitch thread is 76 dtex (10 g/m^2^). The matrix material, supplied from Dost Kimya (İstanbul, Turkey), is composed of a mixture of MGS^®^-LR160 epoxy resin and its hardener, MGS^®^-LH160. As specified by the supplier, the density and the viscosity of the MGS^®^-LR160 epoxy resin at 25 °C are 1.13–1.17 g/cm^3^ and 700–900 mPas, respectively. Additionally, the density and the viscosity of the MGS^®^-LH160 hardener at 25 °C are 0.96–1.00 g/cm^3^ and 10–50 mPas, respectively. The resin-to-hardener mixing ratio per weight is 100:25. The resin exhibits a tensile strength ranging from 70 to 80 MPa, a flexural strength between 110 and 140 MPa, and a modulus of elasticity in the range of 3.2 to 3.5 GPa. Its glass transition temperature lies between 75 °C and 80 °C [32].

In this study, SSWWM (AISI 304) was used as the patch material. It is widely utilised as a reinforcement material due to its significant properties, such as superior mechanical and thermal properties and lightweight [23]. The chemical composition of AISI 304 austenitic stainless-steel is as follows: 0.045% C, 18.13% Cr, 8.25% Ni, 0.053% N, 0.44% Si, 1.11% Mn, 0.0076% S and 0.034% P (mass%) [33]. Some of the mechanical properties of the AISI 304 stainless-steel are illustrated in Table 1. In this study, four types of SSWWMs with mesh counts of 20, 40, 60, and 80 were used as the patch material. Here, the mesh count refers to the number of meshes per inch. A comprehensive description of the SSWWMs employed in this study can be found in Ref. [15].

Loctite-EA 9466 epoxy adhesive, produced by Henkel Company (Düsseldorf, Germany) [35], was used for bonding the patch material to the parent laminate. It is a two-component, epoxy-based adhesive that offers superior bonding strength for a wide variety of plastics and metals [35]. The mechanical characteristics of Loctite-EA 9466 adhesive, obtained through uniaxial tensile testing conducted at room temperature (22 °C) by Rahmani and Choupani [36], are presented in Table 2.

### 2.2. Fabrication of the Parent Laminate

**Figure 1:**
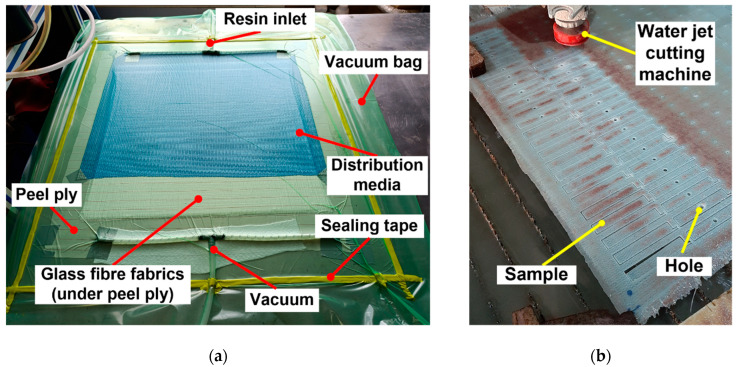
Fabrication of the parent laminate. (**a**) Application of VARI method. (**b**) Water jet cutting.

The vacuum-assisted resin infusion (VARI) method was utilised to manufacture the parent laminate. The application of the VARI method is shown in Figure 1a. In this method, initially, the mold was thoroughly cleaned with a cloth to eliminate any dust or contaminants. Subsequently, a release film was placed over the mold. Six layers of unidirectional E-glass fabric, each measuring 750 mm×750 mm, were placed over the release film. A peel ply, which enables a smooth upper surface, was then placed over the fabric layers. Resin distribution media was positioned on top of the peel ply, and then T-connectors and spiral tubes were installed to direct the resin flow. Finally, the whole system was sealed with a vacuum bag, and a vacuum pressure of 0.1 MPa was applied to remove air from the system. After the air evacuation, the MGS^®^-LR160/MGS^®^-LH160 mixture was drawn into the system until all fabrics were fully wet. The resin infusion time was approximately 30 min. The curing process was performed at room temperature (25 °C) for 24 h, during which a stable vacuum pressure of 0.1 MPa was consistently maintained. Then the composite plate was subjected to post-curing at a table temperature of 80 °C for 15 h under constant vacuum pressure. In this way, the parent laminate with a stacking sequence of [0°]_6_ was fabricated. Following the production of the parent laminate, it was cut into the standard dimensions of the 3PB test samples (77 mm×13 mm) by utilising a water jet cutting machine, as illustrated in Figure 1b. Moreover, a circular hole with a diameter of 5 mm was cut at the centre of the parent laminate to simulate the damaged area. When a crack or damage occurs in a structure, a commonly recommended repair method involves removing the damaged region by cutting it in a circular shape [31]. Introducing a hole into the structure can result in stress concentration. However, circular holes are advantageous compared to sharp-cornered geometries such as square or rectangular shapes, as they produce lower stress concentrations due to their reduced stress concentration factor.

### 2.3. Repair of the Parent Laminate

**Figure 2:**
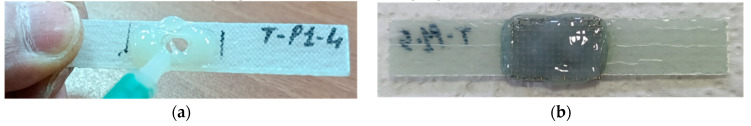

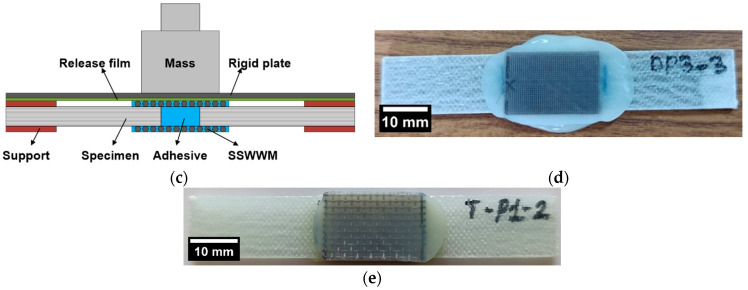
Repair steps of the parent laminate. (**a**) Adhesive application. (**b**) Placing the SSWWM. (**c**) Applying constant pressure during the curing process. (**d**) Sample after completion of the curing process. (**e**) Sample after grinding excess adhesive.

The repair stages of the parent laminate are illustrated in Figure 2. Prior to the implementation of the bonded repair, the SSWWMs were cut to dimensions of 20 mm×13 mm using sheet metal cutting shears. The SSWWMs were then kept in acetone for 15 min to remove any contaminants and dust on their surfaces. Simultaneously, the surfaces of the parent laminates to which the adhesive would be applied were thoroughly cleaned with a cloth wetted with acetone. After a 15 min drying period at room temperature, Loctite-EA 9466 epoxy adhesive was applied to the cleaned surfaces of the parent laminates. Subsequently, SSWWMs were carefully positioned over the adhesive-coated region. A metallic mass weighing 750 g was placed on top of each group of five test samples to ensure constant pressure during 24 h curing process at room temperature (25 °C). To ensure uniform pressure distribution, a rigid plate weighing 150 g was positioned between the metallic mass and the samples. Additionally, a release film was placed between the rigid plate and the samples to prevent unwanted bonding between the samples and the rigid plate. Moreover, to prevent any shifting of the SSWWMs during bonding, supports with the same thickness as the SSWWMs were strategically positioned at the ends of the samples. After the curing process was completed, any excess adhesive protruding beyond the bonding region was removed using a grinder.

The potential corrosion issues of SSWWM are an important consideration for the long-term durability of repaired parent laminates, particularly in aggressive service conditions. The external patch repair method used in this study is typically regarded as a provisional solution intended to reinstate mechanical integrity until a permanent repair can be implemented [37]. In such repair scenarios, the structure is not intended to remain in service for an extended period; thus, evaluation of long-term durability is not applicable. Consequently, the material is unlikely to be exposed for a sufficient duration to initiate or propagate corrosion, rendering it a negligible concern in this context. Furthermore, AISI 304 austenitic stainless-steel was chosen due to its high corrosion resistance, aiming to minimise the risk of corrosion over the intended service period.

**Figure 3:**
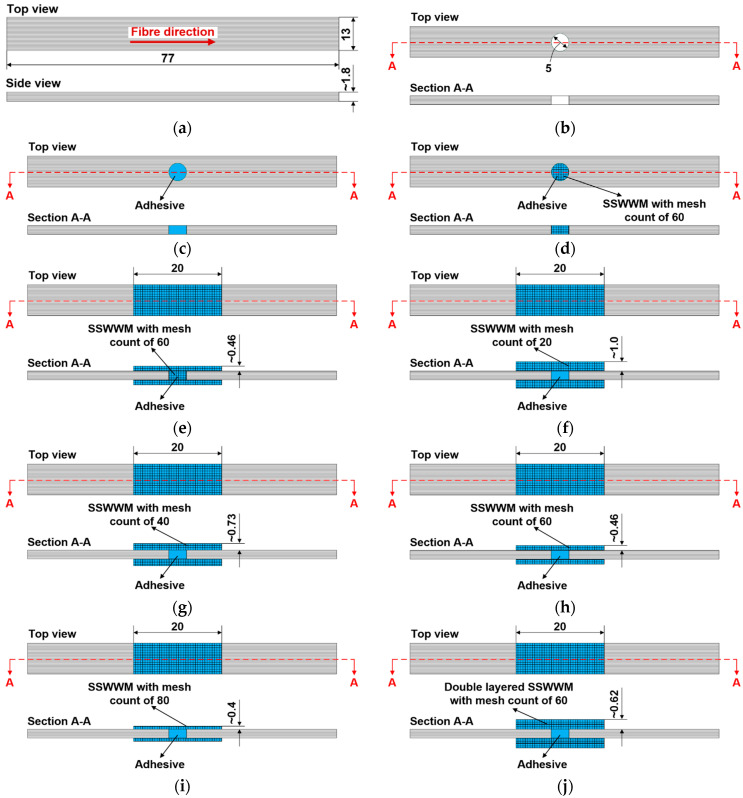
Geometric configurations of test samples (all dimensions are in mm). (**a**) Control sample. (**b**) Hole. (**c**) A plug. (**d**) A/M60 plug. (**e**) A/M60 plug + M60 patch. (**f**) A plug + M20 patch. (**g**) A plug + M40 patch. (**h**) A plug + M60 patch. (**i**) A plug + M80 patch. (**j**) A plug + 2L-M60 patch.

The design details and the geometric configurations of both the repaired and un-repaired 3PB test samples are presented in Table 3 and Figure 3, respectively. Specimen “A plug” was designed to investigate the impact of plugging the open hole with adhesive, while the specimen “A/M60 plug” was developed to examine the effect of plugging the open hole with SSWWM-reinforced adhesive on the flexural behaviour of parent laminate. Specimen “A/M60 plug + M60 patch” was produced to examine the effectiveness of double-sided external patching in repairing a parent laminate. Moreover, “A plug + M20 patch”, “A plug + M40 patch”, “A plug + M60 patch”, and “A plug + M80 patch” specimens were fabricated to investigate the influence of mesh count of SSWWM bonded to both sides of the parent laminate for repair on the flexural behaviour of the parent laminate. Lastly, specimen “A plug + 2L-M60 patch” was produced to evaluate the impact of the number of SSWWM layer on the flexural behaviour of the parent laminate.

The weights of the test samples, measured with a scale with a precision of 0.02 g, are detailed in Table 4. The implementation of the patch resulted in a weight increase ranging from 1.03% to 49.56% when compared to the Control sample. An increase in weight by 49.59% is considered quite significant. However, in this study, the ratio of the damage size to the total specimen size is relatively high. In large-scale structures, damage of such a proportion are typically addressed through component replacement rather than repair. Only minor damage, relative to the overall size of the structure, is generally repaired. Therefore, it is believed that if this method were to be applied to large structures, the resulting weight increase could be negligible.

### 2.4. Three-Point Bending Tests

**Figure 4:**
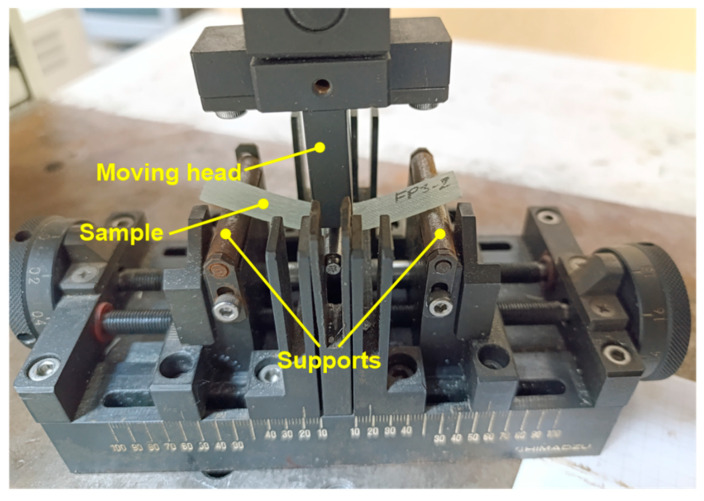
Three-point bending test setup.

3PB tests were conducted in accordance with the ASTM D7264/D7264M-15 standard [38] using a universal testing machine (AG-IS, Shimadzu Corp., Kyoto, Japan) with a load capacity of 5 kN. The machine was outfitted with a 3PB test configuration, as shown in Figure 4. 3PB tests were carried out at room temperature, with a test speed of 2 mm/min. The support span was set at 57 mm. Displacement during testing was recorded based on the crosshead movement of the universal testing machine. To enhance the reliability of the results and minimise potential experimental errors, four replicate samples were tested. As a result of the experimental investigations, comprehensive data regarding force and displacement were obtained.

## 3. Results and Discussion

### 3.1. Statistical Analysis of the Test Results

Statistical analysis of the average test results is conducted to assess whether they are the result of random chance or not. This process is critical for ensuring the reliability and replicability of the research outcomes. Table 5 presents the mean values and standard deviations of the failure loads obtained through 3PB tests, as well as the *p*-values. The *p*-value measures the statistical significance of the average test results. In this work, independent *t*-tests are conducted to calculate *p*-values, which are calculated by using the $“t_{score}”$ value in the T-table. Although the sample sizes were relatively small, approximate normal distribution of the data was assumed. Moreover, all test samples were prepared and tested independently. At a 95% confidence level, the results are considered statistically significant if the *p*-value is less than 0.05.

The *p*-values obtained from the statistical analysis for the A/M60 plug + M60 patch, A plug + M40 patch, and A plug + 2L-M60 patch samples are 0.19, 0.43, and 0.65, respectively, indicating statistical insignificance. These findings indicate that the 3PB failure loads of the “A/M60 plug + M60 patch”, “A plug + M40 patch”, and “A plug + 2L-M60 patch” samples do not differ significantly from the Control sample.

### 3.2. Influence of Plugging the Open Hole

**Figure 5:**
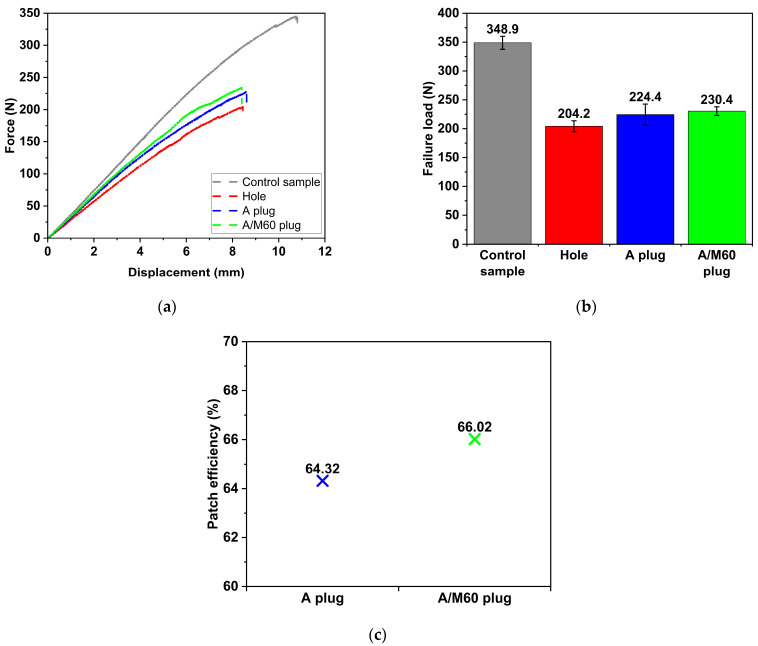
Effect of hole repair (**a**) Force–displacement curves. (**b**) Failure loads. (**c**) Patch efficiencies.

Force–displacement curves of the un-damaged, damaged, and hole-repaired parent laminates, obtained through 3PB tests, are illustrated in Figure 5a. To fully comprehend the behaviour of the repaired samples, it is crucial to first understand the behaviours of both the undamaged (“Control”) and damaged (“Hole”) samples. Both the “Control” and “Hole” samples exhibit almost linear behaviour, transitioning into a nonlinear phase prior to failure. Under bending loading, the samples undergo stresses along the span length. The plies above the neutral surface experience compressive stress, while the plies below the neutral surface experience tensile stress. In this work, the parent laminate with a stacking sequence of [0°]_6_ was fabricated, ensuring that the fibres were aligned along the span of the samples. Therefore, the linear behaviour of the E-glass fibres dominates the behaviours of the “Control” and “Hole” samples. Furthermore, the damaged sample demonstrates reduced stiffness compared to the undamaged sample.

Plugging the open hole significantly affects the force–displacement response of the parent laminate. Although plugging the open hole with adhesive (“A plug” sample) does not substantially alter linear behaviour, when SSWWM with a mesh count of 60 is incorporated into the adhesive (“A/M60 plug” sample), the behaviour becomes nonlinear. This shift in behaviour can be attributed to the nonlinear characteristics of SSWWM. Furthermore, plugging the open hole with adhesive results in an increase in the slope of the force–displacement curve compared to that of damaged (“Hole”) sample. Incorporating SSWWM into the adhesive does not significantly influence the slope of the curve. This is likely due to the relatively small quantity of SSWWM in the adhesive, which does not substantially alter the adhesive stiffness. It is also important to note that the slope of the force–displacement curve for the “Control” sample remains higher than that of the repaired samples, which can be attributed to the lower stiffness of the adhesive relative to that of the parent laminate.

Upon examining the failure loads illustrated in Figure 5b, the presence of damage (“Hole” sample) results in a 41.5% decrease in 3PB failure load relative to the “Control” sample. The presence of a hole diminishes the effective fibre content, resulting in a significant reduction in the load-bearing capacity. The application of adhesive to plug the open hole (“A plug” sample) leads to a 9.9% improvement in the failure load relative to the “Hole” sample. Similarly, Soutis et al. [37] reported that plugging the open hole in the sample mitigates the stress concentration at the hole edges, thereby enhancing the performance. However, this repaired sample still exhibits a failure load that is 35.7% lower than that of the “Control” sample. This phenomenon can be attributed to the disparity in stiffness between the patch material and the parent laminate. This disparity is likely to induce localised stresses within the patch region and hinder effective load transfer between the patch and parent laminate. On the other hand, the incorporation of SSWWM into the adhesive (“A/M60 plug” sample) results in only an insignificant increase of 2.7% in the failure load compared to the “A plug” sample. This indicates that the presence of SSWWM does not significantly enhance the load-bearing capacity under 3PB loading conditions. As for failure displacement, the presence of a hole in the sample significantly decreases the failure displacement compared to the “Control” specimen. Although filling the hole with adhesive (“A plug” sample) and further reinforcing it with SSWWM (“A/M60 plug” sample) introduces slight changes, these changes remain negligible when compared to the failure displacement of the holed sample.

Patch efficiencies of the repaired samples are presented in Figure 5c. The patch efficiency is calculated by dividing the failure load of the repaired sample by that of the “Control” sample and multiplying the result by 100. This metric quantifies the capability in restoring the failure load of the repaired sample to its undamaged state. As seen in Figure 5c, the restored failure loads are 64.32% and 66.02% of the “Control” sample for “A plug” and “A/M60 plug” samples, respectively.

### 3.3. Influence of the External Patch Repair

**Figure 6:**
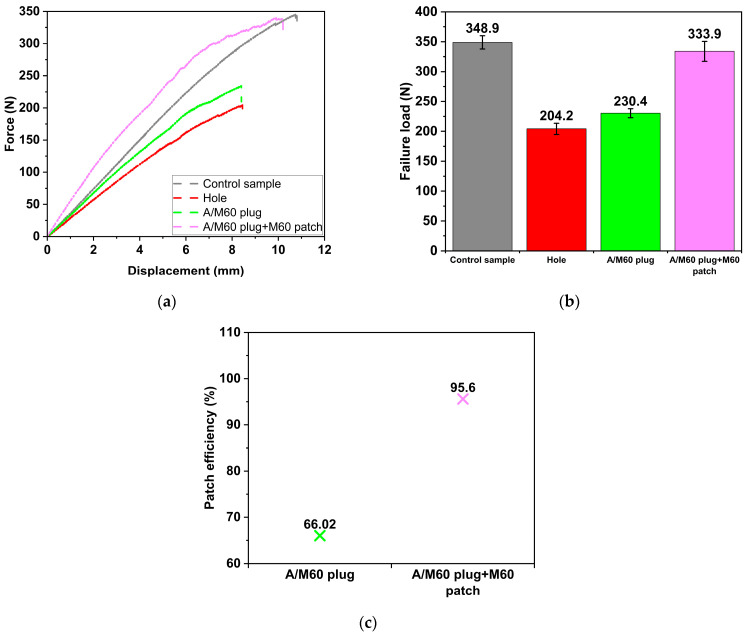
Effect of external patch repair. (**a**) Force–displacement curves. (**b**) Failure loads. (**c**) Patch efficiencies.

To evaluate the impact of the external patch repair on the 3PB behaviour of the parent laminate, SSWWM with a mesh count of 60 was adhesively bonded to both sides of the damaged region of the “A/M60 plug” sample. As seen in Figure 6a, the external patch repair significantly influences the force–displacement characteristics of the parent laminate. The application of the external patch leads to a noticeable increase in the slope of the force–displacement curve compared to the “Control” specimen. This enhancement is likely due to the higher stiffness of the SSWWM compared to the parent laminate. A similar observation was reported by Karunagaran and Rajadurai [39], who investigated the mechanical behaviour of stainless-steel wire mesh/glass fibre epoxy hybrid composites and concluded that the incorporation of stainless-steel wire mesh significantly improved the composite’s stiffness. Furthermore, the patch may provide additional restraint against local deformations, thereby contributing to the overall enhancement in bending stiffness. Moreover, in the case of the external patch implementation, nonlinearity is more evident in comparison to the “A/M60 plug” sample. This behaviour may be attributed to the nonlinear behaviour of SSWWM.

In addition, as illustrated in Figure 6b, the external patch enhances the load-bearing capacity of the “A/M60 plug” sample by 44.9%. The observed rise in the failure load of the “A/M60 plug + M60 patch” sample, compared to the “A/M60 plug” sample, can be attributed to the enhanced structural integrity provided by the external patch, which facilitates stress redistribution. The introduction of an external patch with different mechanical properties relative to the parent laminate leads to a modification in the local mechanical properties of the parent laminate. The external patch effectively distributes the applied load over a broader area, thereby reducing the localised stress at the hole edge. This reduction in localised stress prevents the crack from spreading further [40]. Moreover, it is important to emphasise that the failure load of the “A/M60 plug + M60 patch” sample is 4.3% lower than that of the “Control” sample.

The patch efficiencies of the “A/M60 plug” and “A/M60 plug + M60 patch” samples are compared in Figure 6c. The external patch repair recovers 95.6% of the undamaged 3PB load-bearing capacity, whereas plugging the open hole with SSWWM-reinforced adhesive (“A/M60 plug” sample) only restores 66.02% of the undamaged 3PB load-bearing capacity. In this regard, it is evident that the application of external patches can restore the load-carrying capacity to a level nearly identical to that of the undamaged sample.

### 3.4. Influence of the SSWWM Count

**Figure 7:**
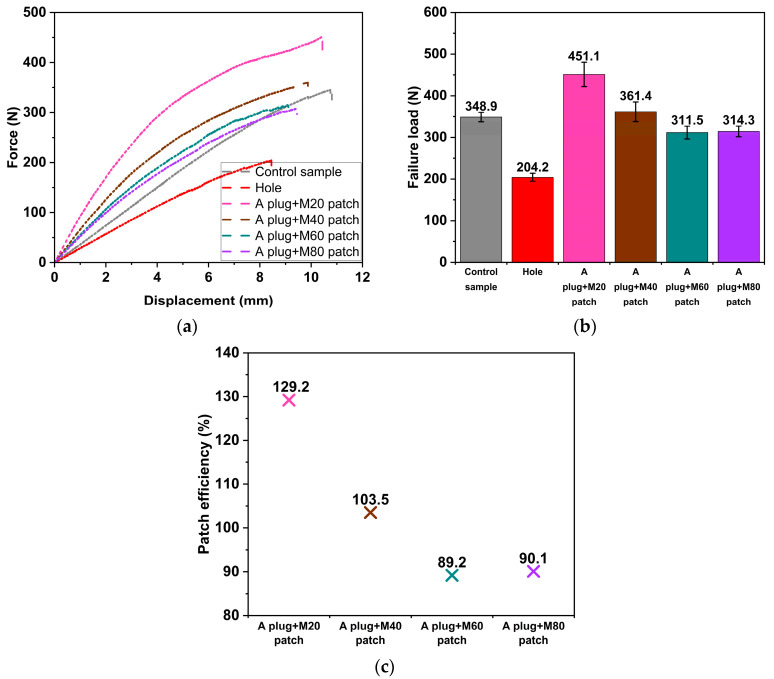
Effect of SSWWM count (**a**) Force–displacement curves. (**b**) Failure loads. (**c**) Patch efficiencies.

The mesh count of the SSWWM used for external patch repair is a key factor influencing the 3PB performance of the parent laminate. As illustrated in Figure 7a, a decrease in the mesh count leads to an increase in the slope of the force–displacement curve. As the mesh count decreases, the wire diameter of the SSWWM increases, resulting in a thicker SSWWM and a greater patch thickness. A thicker SSWWM exhibits higher bending rigidity compared to a thinner SSWWM. This increased rigidity is likely to cause an increase in the slope of the force–displacement curve. Furthermore, changes in mesh count not only affect the slope of the force–displacement curve but also influence the characteristics of the force–displacement curve. Regarding the mesh count’s influence on the force–displacement curve characteristics, all repaired parent laminates exhibit nonlinear behaviour, and nonlinearity becomes more pronounced as the mesh count decreases.

Figure 7b compares the damage loads of the repaired parent laminates, considering the effect of SSWWM count. The failure loads of the parent laminates, which were repaired using SSWWM with mesh counts of 20, 40, 60, and 80, are 451.1 N, 361.4 N, 311.5 N, and 314.3 N, respectively. The failure load clearly diminishes as the mesh count increases within the range of 20 to 60 mesh count. The reduction in failure load can be attributed to the decrease in wire diameter. As previously mentioned, an increase in the mesh count corresponds to a reduction in the wire diameter of SSWWM. The efficiency of load transfer from the matrix to the fibres is highly dependent on the fibre–matrix interface quality [41]. The lack of surface treatment on the SSWWM wires used in this study may lead to insufficient adhesion between the wires and the adhesive. Moreover, wires with smaller diameters offer a greater surface area, resulting in more defects at the interface, and therefore reduced failure load. Additionally, as noted by Aabid et al. [42], a thicker patch enhances the load-bearing capacity and enables more effective load distribution, thereby mitigating stress concentrations at the crack tip. As previously discussed, a reduction in the mesh count leads to an increase in patch thickness, which may have further contributed to the observed improvement in failure load. However, further increasing the mesh count does not influence the failure load. Beyond a certain mesh count of 60, the corresponding increase in interface defects reaches a threshold where additional mesh increments no longer significantly impact the overall load-bearing capacity.

As shown in Figure 7c, for parent laminates repaired with SSWWM of various mesh counts, the patch efficiency ranges from 89.2% and 129.2%. The parent laminate repaired with SSWWM with a mesh count of 20 exhibits superior performance compared to the “Control” sample. This phenomenon can be attributed to the increased bending rigidity resulting from the greater thickness of the SSWWM with a mesh count of 20. Greater bending rigidity leads to increased resistance against deformation, requiring higher applied forces for bending to occur. Furthermore, the patching procedure effectively mitigates stress concentration effects at the edges of the hole, which in turn enhances the load-bearing capacity of the repaired parent laminate. By reducing localised stress intensities, the patch delays the initiation and propagation of damage, thereby contributing to a higher failure load. The patch efficiency exhibits a clear decline as the mesh count increases within the range of 20 to 60 mesh count. However, beyond this range, further increases in the mesh count do not significantly affect patch efficiency.

### 3.5. Influence of the Number of SSWWM Layers

**Figure 8:**
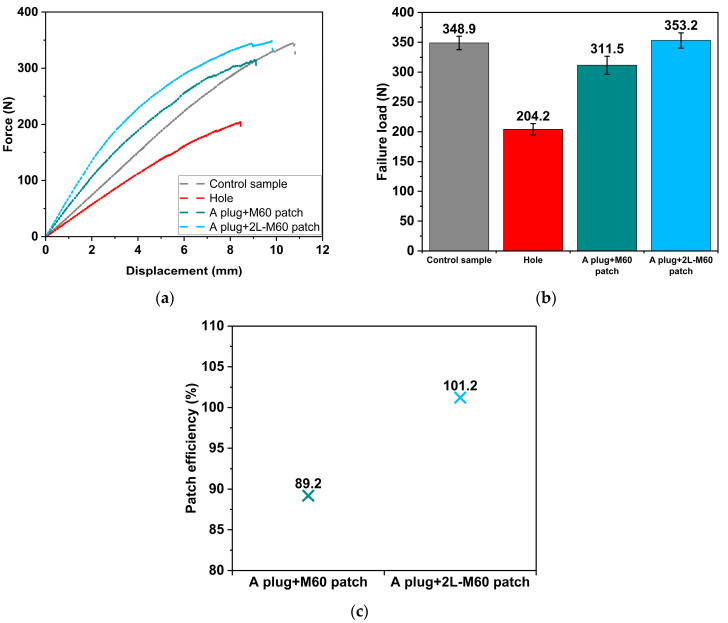
Effect of number of SSWWM layer. (**a**) Force–displacement curves. (**b**) Failure loads. (**c**) Patch efficiencies.

Force–displacement curves illustrating the impact of number of SSWWM layers on the 3PB characteristics of parent laminate are presented in Figure 8a. The number of SSWWM layers plays a significant role in the 3PB behaviour of the parent laminate. When the number of SSWWM layers increases from one to two, the slope of the force–displacement curve becomes steeper. This increase can be attributed to the increase in the bending rigidity of the external patch due to the stacking of SSWWMs.

In addition, as illustrated in Figure 8b, the number of SSWWM layers has a major influence on the load-bearing capacity of the parent laminate. Increasing the SSWWM layer from one to two boosts the failure load by 13.4%. This improvement may be ascribed to the increase in patch thickness. The increase in patch thickness improves the patch’s ability to transfer applied loads effectively between the parent laminate and patch [42]. It is also believed that mechanical interlocking between the stacked SSWWMs improves the structural integrity and the load transfer between the patch and the parent laminate. However, as emphasised by Aabid et al. [42], it is essential to prevent the patch from becoming excessively stiff, as this may lead to a greater stress concentration at the patch edges, potentially resulting in failure at these locations. In addition, excessive stiffness can create a significant mismatch in the stiffness distribution between the patch and the parent laminate, disrupting the smooth transfer of loads. Furthermore, an excess increase in patch thickness may reduce the failure load due to the increased shear stress at the patch/parent laminate interface. Therefore, it is believed that there is an optimal patch thickness that maximises the load-bearing capacity without introducing excessive stress concentrations or interface shear stresses, balancing the benefits of improved load transfer and structural integrity.

The analysis of the impact of the number of SSWWM layers on patch efficiency (Figure 8c) reveals that a 101.2% recovery of the failure load is achieved when the parent laminate is repaired with two layers of SSWWM. This indicates that the 3PB performance of the parent laminate repaired with two layers of SSWWM is equivalent to that of the “Control” sample.

### 3.6. Damage Modes

**Figure 9:**
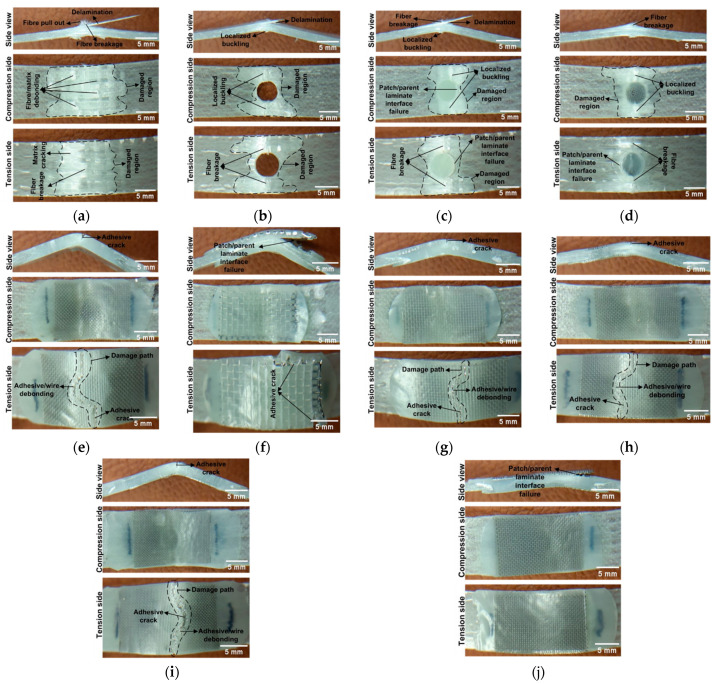
Failure modes of the (**a**) Control sample, (**b**) hole, (**c**) A plug, (**d**) A/M60 plug, (**e**) A/M60 plug + M60 patch, (**f**) A plug + M20 patch, (**g**) A plug + M40 patch, (**h**) A plug + M60 patch, (**i**) A plug + M80 patch, and (**j**) A plug + 2L-M60 patch.

To fully understand the impact of patch repair, it is essential to analyse the failure modes of the samples. Figure 9 depicts the failure modes of the un-damaged, damaged, and repaired samples. Under flexural loading, the region above the neutral axis (compression side) of the specimen undergoes compressive stress, whereas the region below the neutral axis (tension side) experiences tensile stress. Upon examining the failure modes of the undamaged sample (“Control” sample) (Figure 9a), several damage mechanisms are observed, including delamination, fibre breakage, fibre/matrix debonding, matrix cracking, and fibre pull-out. Furthermore, the damage observed in the un-damaged sample spread over a wide area instead of being concentrated at a point, causing its strength to be high. When analysed the damaged surfaces of the damaged sample (“Hole” sample) (Figure 9b), delamination, localised buckling which is not observed in the “Control” sample, and fibre breakage are evident. Additionally, due to stress concentration, damage is localised in a small region surrounding the hole, in contrast to the “Control” sample where stress is more evenly distributed over a broader area. This localised damage provides a clear explanation for the reduction in failure load observed in comparison to the “Control” sample. It is also believed that the localised buckling observed on the compression side is induced by this stress concentration. Delamination, fibre breakage, and localised buckling are also observed in the “A plug” and “A/M60 plug” samples (Figure 9c,d). The occurrence of similar failure modes in both samples is the clear indication of the negligible difference in fracture load. Moreover, failure at the patch/parent laminate interface occurs. The discrepancy in flexural stiffness between the parent laminate and the patch may have led to additional stresses at the parent laminate/patch interface, potentially resulting in damage at this interface. These observations further explain that the fracture loads of the “A plug”, “A/M60 plug”, and “Hole” samples are comparable, as all exhibit stress concentrations contributing to similar failure behaviour. As for “A/M60 plug + M60 patch” sample (Figure 9e), adhesive crack and adhesive/wire debonding, which are not observed in the “A/M60 plug” sample, are detected at the tension side of the sample. Upon examining the damage path, it is evident that the damage follows a curved path rather than a straight path along the width of the sample. The damage path follows the edge of the hole, likely due to the additional stresses at the parent laminate/patch interface in the hole. In the “A plug + M20 patch” sample (Figure 9f), the failure occurs at the external patch/parent laminate interface, possibly due to the high patch thickness. The occurrence of damage at this interface rather than at the adhesive–wire interface is the clear indication of strong bonding between adhesive and SSWWM, which offers a rational explanation for the high load-bearing capacity of the “A plug + M20 patch” sample. This suggests that, at the point of failure, the patch itself was able to bear significant loads. As highlighted by Aabid et al. [42], increases in the patch thickness must be carefully managed to prevent excessive stiffness, as this can cause stress concentrations at the edges of the patch, potentially leading to failure at these locations. On the other hand, no significant differences are observed in the failure modes of the samples labelled “A plug + M40 patch”, “A plug + M60 patch”, and “A plug + M80 patch” (Figure 9g–i). In all cases, the failure is characterised by adhesive cracking and adhesive/wire debonding. Adhesive/wire debonding is a clear indication of the weak bond between the wires and the adhesive due to the lack of surface treatment on the SSWWM wires. Specifically, surface treatments such as sandblasting, electro dissolution, acid etching, or the application of aryl diazonium grafting and silane grafting have been suggested in the literature to enhance adhesion between adhesive and metal mesh by increasing surface roughness or promoting chemical bonding [39,43,44]. Additionally, the damage path exhibits a similar trend for all samples. Lastly, as for “A plug + 2L-M60 patch” sample (Figure 9j), failure is observed at the interface between the external patch and the parent laminate, likely resulting from the increased stiffness and thickness of the patch. The presence of interfacial damage between the patch and the parent laminate indicates strong mechanical interlocking between the stacked SSWWMs, which enhances both structural integrity and load transfer efficiency. This improved load transfer mechanism accounts for the higher failure load observed in comparison to the “A plug + M60 patch” configuration.

## 4. Conclusions

In this work, the effect of employing SSWWM as a patch material to repair the damaged GFR composite laminate was investigated experimentally through 3PB testing. The influence of plugging the open hole, external patch repair, SSWWM count, and number of SSWWM layers on the 3PB behaviour of damaged parent laminate were examined.

The findings reveal that external patch repair using SSWWM is significantly more effective than simple open-hole plugging. The application of adhesive to plug the open hole (“A plug” sample) resulted in a 9.9% increase in the failure load compared to the damaged sample (“Hole” sample). However, the failure load of the “A plug” sample reached only 64.32% of the failure load observed in the “Control” sample. Reinforcement of the adhesive with SSWWM (“A/M60 plug” sample) did not lead to notable improvement, indicating the limited effectiveness of this repair method.

In contrast, the external patch repair strategy provided considerably higher recovery in mechanical performance. The SSWWM count was found to play a critical role in the 3PB failure load of the repaired laminate. An inverse relationship was observed between mesh count (in the range of 20–60) and 3PB failure load, while higher mesh counts beyond this range did not further affect performance. Patch efficiency for different mesh counts ranged between 89.2% and 129.2%, indicating that, in some cases, the patched samples exhibited higher failure loads compared to the “Control” specimen. Additionally, an increase in the number of SSWWM layers led to a corresponding rise in the failure load. For instance, when the number of SSWWM layers increased from one to two, the failure load increased by 13.4%.

Regarding repair mechanisms, the mesh count of SSWWM appears to influence load transfer efficiency, bending rigidity, and defects at the wire/adhesive interface of the repaired parent laminate, contributing to the increased load-bearing capacity.

In addition, the use of SSWWM as a patch material notably influences the force–displacement characteristics of the parent laminate. While the “Control” and “Hole” specimens demonstrate an approximately linear response followed by a nonlinear behaviour preceding failure, the force–displacement curve of the parent laminate repaired with SSWWM shifts from an almost linear response to a nonlinear response.

The limitations of this study include the restricted mesh count range (20–80) and the focus on quasi-static 3PB without long-term durability or environmental exposure considerations. Future work should explore fatigue behaviour, hygrothermal effects, and in-field applicability of the SSWWM patching system.

Consequently, it has been observed that employing SSWWM as an external patch material enables the desired mechanical properties and damage resistance to be achieved without the need to change the entire structure.

## Figures and Tables

**Table 1:** Mechanical properties of AISI 304 stainless-steel [34].

Properties	Unit	Value
Tensile strength	MPa	515
Yield strength	MPa	205
Young’s modulus	GPa	193
Poisson’s ratio	-	0.27–0.30

**Table 2:** Mechanical properties of Loctite EA-9466 adhesive [36].

Properties	Unit	Value
Young’s modulus	GPa	1.91
Yield strength	MPa	41.33
Ultimate tensile strength	MPa	44.38
Poisson’s ratio	-	0.35
Fracture strain	%	3.8582
Critical strain energy release rate in mode I	J/m^2^	313.76
Critical strain energy release rate in mode II	J/m^2^	155.88

**Table 3:** Design details of test samples.

Sample Code	Description
Control sample	Sample with no damage
Hole	Sample with a 5 mm diameter hole at its centre
A plug	The sample was repaired by filling the hole with adhesive.
A/M60 plug	The sample was repaired by filling the hole with SSWWM-reinforced adhesive. Here, three layers of SSWWM, with a mesh count of 60, were applied.
A/M60 plug + M60 patch	SSWWMs with a mesh count of 60 were bonded to both sides of the specimen “A/M60 plug”.
A plug + M20 patch	The sample was repaired by bonding the SSWWM with a mesh count of 20 to both sides of the sample. Here, the hole was filled solely with adhesive.
A plug + M40 patch	The sample was repaired by bonding the SSWWM with a mesh count of 40 to both sides of the sample. Here, the hole was filled solely with adhesive.
A plug + M60 patch	The sample was repaired by bonding the SSWWM with a mesh count of 60 to both sides of the sample. Here, the hole was filled solely with adhesive.
A plug + M80 patch	The sample was repaired by bonding the SSWWM with a mesh count of 80 to both sides of the sample. Here, the hole was filled solely with adhesive.
A plug + 2L-M60 patch	The sample was repaired by bonding the two layers of SSWWM, each with a mesh count of 60, to both sides of the sample. Here, the hole was filled solely with adhesive.

**Table 4:** Weights of the test samples.

Specimen	Weight (g)	Change (%)
Control sample	2.93 ± 0.02	---
Hole	2.84 ± 0.02	−2.94
A plug	2.96 ± 0.03	1.03
A/M60 plug	2.96 ± 0.03	1.23
A/M60 plug + M60 patch	3.60 ± 0.08	22.97
A plug + M20 patch	4.38 ± 0.10	49.56
A plug + M40 patch	3.94 ± 0.04	34.52
A plug + M60 patch	3.59 ± 0.08	22.62
A plug + M80 patch	3.57 ± 0.04	21.87
A plug + 2L-M60 patch	3.99 ± 0.04	36.50

**Table 5:** Statistical evaluation of the “3PB failure loads (N)” of all specimens.

Sample	Mean	Std. Dev	*p*-Value
Control sample	348.9	11.2	---
Hole	204.2	9.5	<0.001
A plug	224.4	18.3	<0.001
A/M60 plug	230.4	7.5	<0.001
A/M60 plug + M60 patch	333.9	16.7	0.19
A plug + M20 patch	451.1	29.2	<0.001
A plug + M40 patch	361.4	23.4	0.43
A plug + M60 patch	311.5	15.2	0.02
A plug + M80 patch	314.3	12.6	0.006
A plug + 2L-M60 patch	353.2	12.9	0.65

## Data Availability

The original contributions presented in this study are included in the article. Further inquiries can be directed to the corresponding author(s).

## Abbreviations

The following abbreviations are used in this manuscript:

SSWWM	Stainless-steel woven wire mesh.
GFR	Glass fibre-reinforced.
3PB	Three-point bending.
a	Depth.
t	Thickness.
SLJ	Single-lap joint
OSB	Oriented Strand Board.
UD	Unidirectional.
VARI	Vacuum-assisted resin infusion.
